# Risk factors associated with surgical intervention in childhood pleural tuberculosis

**DOI:** 10.1038/s41598-021-82936-4

**Published:** 2021-02-04

**Authors:** Ming Zhou, Shi-Feng Ren, Huai-Zheng Gong, Mao-Shui Wang

**Affiliations:** 1 Department of Central Lab, Longtan Hospital of Guangxi Zhuang Autonomous Region, Liuzhou, Guangxi China; 2 Department of Laboratory Medicine, Xingming Hospital, Xiangcheng, Lanling, Shandong China; 3 Department of Laboratory Medicine, Shandong Provincial Chest Hospital, Cheeloo College of Medicine, Shandong University, 46# Lishan Road, Jinan, 250013 Shandong China

**Keywords:** Tuberculosis, Respiratory distress syndrome, Risk factors, Respiratory distress syndrome

## Abstract

Surgical intervention use is common in the management of childhood pleural tuberculosis (TB), however, its associated risk factors remain unclear. Between January 2006 and December 2019, consecutive children patients (≤ 15 years old) who had a diagnosis of pleural TB were included for the analysis. Surgical intervention was defined as debridement (such as breaking loculations), decortication, and thoracic surgery (such as lobectomy or segmental resection). Patients undergoing surgery were included as surgical group, without surgery were classified as non-surgical group, surgical risk factors were then estimated. Univariate and multivariate logistic regression analysis were performed to evaluate the risk factors for surgical interventions. A total of 154 children diagnosed as pleural TB (definite, 123 cases; possible, 31 cases) were included in our study. Of them, 29 patients (18.8%) were classified as surgical group and 125 patients (81.2%) were classified as non-surgical group. Surgical treatments were analyzed in 29 (18.8%) patients, including debridement (n = 4), decortication (n = 21), and thoracic surgery (n = 4). Further multivariate analysis revealed that empyema (age- and sex-adjusted OR = 27.3, 95% CI 8.6, 87.1; *P* < 0.001) and frequency of hospitalization (age- and sex-adjusted OR = 1.53, 95% CI 1.11, 2.11; *P* < 0.01) were associated with the use of surgical interventions in children with pleural TB. In China, surgical interventions are still required in a significant proportion of children with pleural TB, and the surgical risk is found to be associated with the frequency of hospitalization and empyema. These findings may be helpful to improve the management of children with pleural TB and minimize the risk of poor outcomes.

## Introduction

Tuberculosis (TB) remains one of the most common infectious agents causing death in children. It is estimated that the inpatient case-fatality rate for pneumonia associated with TB ranged from 4 to 21%^1^. In addition, a global mortality burden was calculated at 239,000 in childhood TB, according to a mathematical model^2^. As reported in a previous study, pleural involvement was ranked as the second site affected by TB among childhood TB^3^. Due to the paucibacillary nature of pleural samples, or the disease, the diagnosis of childhood pleural TB remains a difficulty to be defined^4^. Hence, the diagnostic or treatment delay easily occurs and a poor outcome may be then produced. For example, in a previous study, we found the delay in the treatment of pleural TB had an association with empyema^5^.

In general, surgical intervention remains a useful tool in the management of childhood pleural TB. For example, surgical decortication was used for the treatment of pleural empyema, its success rate (96.3%) was higher than those treated with simple drainage (58.3%, *P* < 0.0001)^6^. If loculations are present in the pleural space, surgical treatment may be employed to break them and removal of all exudates^7^. In addition, surgery is also thought as an appropriate treatment for pulmonary and pleural TB^8,9^, especially complicated TB (such as parenchymal destruction and empyema)^10^, and drug-resistant TB with a negative response to medical treatment^11^.

To make a better management of childhood pleural TB, our study aimed to review the surgical interventions employed for the disease and its associated risk factors were also investigated. The findings discovered by the retrospective study may aid to shorten the time spent deciding whether surgery is indicated or not, and may be helpful to improve the current knowledge of the treatment of childhood pleural TB.

## Materials and methods

The study done by me and my colleagues was approved by the Ethics Committee of Shandong Provincial Chest Hospital (No. 2020XKYYEC-29). Due to the retrospective nature of the study design and anonymous data collection, written informed consent was waived. The study was performed at the Department of Lab Medicine, Shandong Provincial Chest Hospital in compliance with Helsinki declaration. Shandong province locates at the eastern part of China, with a population of > 90 million people. In 2018, nearly 30,000 new TB cases were reported and about 200,000 cases were registered as active TB. The Shandong Provincial Chest Hospital is a provincial referral TB hospital, with approximately 1000 beds. Every year, about 3000–5000 TB cases are admitted to our hospital for treatment.

Between January 2006 and December 2019, consecutive children patients (≤ 15 years old) who had a diagnosis of pleural TB were included for the analysis. Pleural TB was defined if one of the following criteria was met: (1) definite: positive mycobacterial culture, or TB RT-PCR (sputum, pleural effusion, or pleural tissue), or suggested by pathological evidences (such as caseous necrosis, or Langhans' giant cells); (2) possible: clinical symptoms and positive acid-fast bacilli (AFB) smear. Other TB such as pulmonary TB, bronchial TB, and tuberculous lymphadenitis were defined by the site of involvement of TB. The demographic, clinical, laboratory, and radiographic data were collected from the electronic medical records retrospectively. A hospitalization was defined as an illness episode resulting in overnight admission to a hospital for treatment. Treatment delay was defined as the interval between symptom initiation and anti-TB therapy. All lab tests were performed within one week after admission.

Surgical intervention was defined as debridement (such as breaking loculations), decortication, and thoracic surgery (such as lobectomy or segmental resection). Decortication was defined as an invasive procedure to remove thickened pleura and surgical debridement was performed to clean the wound bed by removing all debris, slough, and obvious evidence of infection. Patients undergoing surgery were included as surgical group, without surgery were classified as non-surgical group, and then surgical risk factors were estimated in the study. Chest radiographs such as X-ray, CT, or ultrasound, were used to evaluate the lesions. Patients were followed up until discharge. All patients were administered with first line anti-TB drugs (isoniazid, rifampicin, ethambutol, and pyrazinamide) for nine months.

SPSS version 16.0 (SPSS, Chicago, IL, USA) was employed for statistical analysis. Continuous variables were presented as mean ± standard deviation (SD) and categorical variables as count (percentages). Univariate logistic regression analysis was performed to evaluate the risk factors for surgical intervention. All variables with a *P* value of < 0.05 in the univariate analysis were included in the final multivariate logistic regression analysis. When applicable, the corresponding odds ratios (OR) and 95% confidence interval (CI), adjusted by age and sex were reported^5^. In addition, to make a better clinical understanding, continuous variables were transformed into categorical variables according to the receiver operating characteristic curve (ROC) analysis. The accuracy of the multivariate model was assessed using the Hosmer–Lemeshow goodness-of-fit test. A *P* value < 0.05 was considered significant.

## Results

### Patients characteristics

A total of 154 children diagnosed as pleural TB (definite, 146 cases; possible, 8 cases) were included in our study. Of them, 29 patients (18.8%) were classified as surgical group and 125 patients (81.2%) were classified as non-surgical group. The average of age was 12.4 ± 3.3 years, weight was 46.1 ± 16.2 kg, 100 (64.9%) of them were male, and 93 (60.4%) of them were from rural areas. One hundred and three patients were tested for HIV status and all of them were HIV-negative. The demographic and clinical characteristics of the eligible patients were presented in Table 1 and Supplementary Table S1.

**Table 1:** Univariate analysis of the demographic data associated with surgical intervention in childhood pleural TB.

N	Total (n)	Surgical group (n)	Non-surgical group (n)	*P* value	OR (95% CI)
	154	29	125		
**Demographic characteristics**					
Age (years)	12.4 ± 3.3	12.8 ± 3.0	12.3 ± 3.4	0.403	
Sex (male)	100 (64.9%)	21 (72.4%)	79 (63.2%)	0.351	
Weight (Kg)	46.1 ± 16.2	47.6 ± 16.5	45.8 ± 16.2	0.591	
Rural area	93 (60.4%)	20 (69.0%)	73 (58.4%)	0.297	
**Medical history**					
Contact history of TB	20 (13.0%)	2 (10.5%)	18 (14.4%)	0.290	
Transferred times	2.1 ± 1.0	2.0 ± 0.8	2.1 ± 1.1	0.481	
Transferred from a teaching hospital	91 (59.1%)	18 (62.1%)	73 (58.4%)	0.717	
Frequency of hospitalization	2.0 ± 1.6	3.2 ± 2.4	1.7 ± 1.2	0.000	1.617 (1.281, 2.042)
Treatment delay (days)	61.8 ± 134.6	58.5 ± 68.8	62.5 ± 145.9	0.885	
Loculated effusion	27	8 (27.6%)	19 (15.2%)	0.120	
**Symptoms**					
Cough	84 (54.5%)	13 (44.8%)	71 (56.8%)	0.246	
Fever	134 (87.0%)	25 (86.2%)	109 (87.2%)	0.886	
Chest pain	69 (44.8%)	13 (44.8%)	56 (44.8%)	0.998	
Dyspnea	42 (27.3%)	7 (24.1%)	35 (28.0%)	0.674	
Sputum production	29 (18.8%)	4 (13.8%)	25 (20.0%)	0.444	
Cavity	7 (4.5%)	1 (3.4%)	6 (4.8%)	0.754	
Empyema	27(17.5%)	19 (65.5%)	8 (6.4%)	0.000	27.787 (9.739, 79.288)
**Clinical chemistry (serum)**					
Total protein (g/L)	68.7 ± 6.9	70.6 ± 7.2	68.3 ± 6.8	0.116	
Albumin (g/L)	38.8 ± 4.8	40.1 ± 3.9	38.5 ± 4.9	0.122	
Blood urea nitrogen (mmol/L)	3.9 ± 1.2	3.7 ± 1.1	3.9 ± 1.2	0.520	
Creatinine (μmmol/L)	52.3 ± 15.3	59.0 ± 13.0	50.8 ± 15.4	0.014	1.036 (1.007, 1.066)
Glucose (mmol/L)	4.8 ± 1.2	4.7 ± 0.6	4.9 ± 1.3	0.560	
Lactate dehydrogenase (U/L)	219.8 ± 71.1	202.0 ± 51.5	224.5 ± 75.0	0.171	
Erythrocyte sedimentation rate (mm/h)	40.8 ± 26.4	31.1 ± 26.3	43.0 ± 26.1	0.039	0.980 (0.962, 0.999)

*TB* tuberculosis, *OR* odds ratio, *CI* confidence interval.

Surgical treatment was given to 29 (18.8%) patients, including debridement (n = 4), decortication (n = 21), and thoracic surgery (n = 4). Twenty (13.0%) patients had a TB contact history, 89 (57.8%) patients of them had pulmonary TB, 13 (8.4%) had tuberculous lymphadenitis, 7 (4.5%) had milliary TB, 4 (2.6%) had tuberculous meningitis, and 3 (1.9%) had bronchial TB. Fever (134, 87.0%) was reported as the most common symptom, followed by cough (84, 54.5%), chest pain (69, 44.8%), dyspnea (42, 27.3%), and sputum production (29, 18.8%). Prior to our center, most of the patients (91, 59.1%) were hospitalized for treatment at a teaching hospital, and the mean frequency of hospitalization were 2.0 ± 1.6.

Among non-surgical cases, a total of 85 cases (68.0%) were treated with aspirated or drained with an inter-costal drain. Among the surgical cases, the corresponding indications for surgery were as follows: no response to anti-TB therapy (n = 6), empyema (n = 19), and persistent breathlessness due to pleural thickening (n = 4). Twenty of them were administrated with anti-TB therapy before surgery, the mean duration was 3.2 months (range 1–10 months). Symptoms were alleviated in all patients before discharge and no death was reported.

Other characteristics, such as radiographic features, lab examinations (blood cell count, chemistry analysis, and flow cytometry analysis), were summarized in Table 1 and Supplementary Table S1. Additionally, all patients undergoing surgery were followed up during their hospital stay, and no significant complications (such as death, recurrence, and ICU stay) were observed.

### Univariate and multivariate analysis

Table 1 shows the results of univariate analysis of a comparison between surgical and non-surgical groups. It was found that the use of surgical interventions were associated with creatinine in serum (OR = 1.036, 95% CI 1.007, 1.066), erythrocyte sedimentation rate (OR = 0.980, 95% CI 0.962, 0.999), frequency of hospitalization (OR = 1.617, 95% CI 1.281, 2.042) and empyema (OR = 27.787, 95% CI 9.739, 79.288; all *P* < 0.05).

Further multivariate analysis (Hosmer–Lemeshow goodness-of-fit test: χ^2^ = 6.46, *df* = 8, *P* = 0.596) revealed that empyema (age- and sex-adjusted OR = 27.3, 95% CI 8.6, 87.1; *P* < 0.001) and frequency of hospitalization (age- and sex-adjusted OR = 1.53, 95% CI 1.11, 2.11; *P* < 0.01) were associated with the use of surgical interventions in children with pleural TB (Table 2).

**Table 2:** Age- and sex-adjusted OR for risk factors associated with surgical intervention in childhood pleural TB.

	Adjusted (age and sex) OR	*P* value
Empyema	27.3 (8.6, 87.1)	< 0.001
Frequency of hospitalization	1.53 (1.11,2.11)	0.009

*TB* tuberculosis, *OR* odds ratio, *CI* confidence interval.

## Discussion

This study presents the clinical characteristics of children who were diagnosed as pleural TB and underwent surgical intervention. Moreover, the risk factors associated with surgical interventions were also evaluated in the study, empyema and frequency of hospitalization were considered as risk factors for the use of surgical treatment. To our knowledge, this study may be the first investigation to assess the surgical risk factors for the management of childhood pleural TB.

First, in terms of the treatment of childhood pleural TB, the proportion of surgical intervention use remains unclear. In general, only a minority would require surgical intervention^12^. However, the accurate level has not been reported. In our study, a significant proportion (18.8%) of children required surgical treatment. The data is comparative with the frequency of surgery in adulthood pleural empyema, which is reported previously^6^. This phenomenon may be contributed to three reasons: (1) regarding pleural TB, compared with the adults in our center, children appear to be more severe and have more cases of empyema (8.9% vs. 17.5%)^5^; (2) the diagnosis of childhood pleural TB remains a challenge, and significant diagnostic delay may occur^13^. Then, a poor condition on admission to hospital may be presented and routine medications may not work in such cases. Therefore, surgical interventions are required to improve their outcome; 3) for childhood pleural TB, the standard treatment remains unknown^14,15^, further research are still in need to explore the efficient treatment for the paucibacillary disease.

Second, the frequency of hospitalization was identified as a risk factor for surgery among children with pleural TB. In our study, the frequency of hospitalization was categorized and described as follows: once (n = 91), twice (n = 32), or more (n = 31). The risk increased significantly by 1.53 (95% CI 1.11, 2.11) per each hospitalization (*P* < 0.01). The frequency was associated with a poor previous treatment and the condition of patient remains unsatisfactory and was required to be improved. The association may be explained by the health service and patient’s status. First, in most areas of China, the role of health services remains limited in the detection of TB^16^, one of the main reasons for this is that few TB assays were performed in most clinical labs, even in some tertiary teaching hospitals. Due to the form of paucibacillary disease, most of TB assays are limited to the detection of pleural TB in children^17^, these challenges make the treatment of childhood pleural TB more complicated. In addition, the disease is relatively neglected in clinical practice as well, and inappropriate treatment may be given. Therefore, the delay in treatment may then contribute a severe presentation on admission to our center and surgical interventions have to be employed for a better outcome of children with the disease. Second, although anti-TB therapy may be given timely. However, it was not always work in all patients with pleural TB, such as empyema and loculated effusion^18^. Therefore, the choice of treatment options should be selected based on the condition of patient. In addition, due to the paucity of studies on pleural TB in children, the treatment strategies are based on adult literature^14,18^, and may be not a feasible strategy.

Third, empyema is another risk factor identified for surgical intervention in the management of childhood pleural TB. This may be explained by the treatment option and insufficient experience in the treatment of tuberculous empyema among children. Currently, the ideal approach for pleural TB in children remains controversial, especially for empyema. On the one hand, the appropriate stage of empyema for surgical treatment required further investigation^19^. For example, Pogorelić et al. found that in the early stage of pleural empyema, video-assisted thoracic surgery could improve the outcome of pediatric patient^20^. In contrast, some authors recommended intrapleural fibrinolytic treatment in patients with stage 1 and 2 empyema^21^. On the other hand, the ideal surgical approach for tuberculous empyema in children also appears unclear. In a systematic review, drainage demonstrated an equal capacity to thoracic surgery^22^. In addition, studies conducted by us and others suggested that, in most children with empyema, antibiotics may result in unnecessary delays in appropriate care and increased hospital charges or frequency of hospitalization^23^. Therefore, most empyemas may require more aggressive treatment, such as decortication and thoracic surgery.

Fourth, as shown in Table 1, other risk factors, such as erythrocyte sedimentation rate (ESR) and creatinine were identified as risk factors in the univariate analysis. However, these variables were not finally included in the multivariable model. The difference in the level of ESR between surgical and non-surgical groups may be associated with empyema, which is known as a serious inflammatory disorder and associated with a lower level of ESR^24^. Similarly, a significant difference in ESR was reported between surgery and drainage groups among patients with pleural empyema^6^. After adjusted for confounding variables, such as sex and age, serum creatinine also was not included in the final model. Interestingly, in a recent study, serum creatinine was identified as a potential biomarker for the diagnosis of pleural TB^25^. However, the accurate mechanism involving the increase of serum creatinine in patients with pleural TB remains unknown.

The study also had several limitations. First, caution should be paid, because the study has a retrospective nature and small sample size. Second, the study was conducted at a single center and the findings may not be proper to generalize to other districts. Third, in the field of pleural TB requiring surgical intervention, although several papers are cited, most of them include only small numbers of children, or only a few studies devoted exclusively to children. The presentation of pleural effusion in the children differs from that in adult by cause, symptom presentation, character of the fluid, techniques for diagnosis, and prognosis^26^. It is, therefore, difficult to generalize these findings observed in adults to children. Fourth, in the study, we aimed to assess the risk factors for surgical treatment. Therefore, diagnostic examinations, such as thoracoscopic examination and thoracentesis were not considered for further analysis. Besides these mentioned above, a larger prospective study is still required to validate our findings.

## Conclusions

Our findings suggest that surgical interventions are still required in a significant proportion of children with pleural TB in China. Further analysis demonstrated that frequency of hospitalization and empyema were associated with the surgical risk in children with pleural TB. These findings stress the characteristics of these patients requiring surgical treatment, then may aid to improve the management of childhood pleural TB and minimize the risk of poor outcomes.

## Supplementary information

Supplementary Information
