# Supplementary material for: Risk factors associated with surgical intervention in childhood pleural tuberculosis
Source: Sci Rep. 2021 Feb 4;11:3084. doi: 10.1038/s41598-021-82936-4 (PMC7862429; doi:10.1038/s41598-021-82936-4)
Supplement: Supplementary file 1 — Supplementary Information [file 41598_2021_82936_MOESM1_ESM.docx]

**Risk factors associated with surgical intervention in childhood pleural tuberculosis**

Ming Zhou^1^, Shi-Feng, Ren^2^, Huai-Zheng Gong^3^, Mao-Shui Wang^3^

^1^ Department of Laboratory Medicine, Longtan Hospital of Guangxi Zhuang Autonomous Region, Liuzhou, Guangxi, China.

^2^ Department of Laboratory Medicine, Xingming Hospital, Lanling, Shandong, China.

^3^ Department of Laboratory Medicine, Shandong Provincial Chest Hospital, Cheeloo College of Medicine, Shandong University, Jinan, Shandong, China.

| Supplementary Table 1. Univariate analysis of the demographic data associated with surgical intervention in childhood pleural TB. | | | | | |
| --- | --- | --- | --- | --- | --- |
|  | | Total (n) | Surgical group (n) | Non-Surgical group (n) | P value |
| N | | 154 | 29 | 125 |  |
| Effusion sites | |  |  |  |  |
|  | Left | 62 (40.3%) | 9(31.0%) | 53(42.4%) | 0.264 |
|  | Right | 74 (48.1%) | 16(55.2%) | 58(46.4) | 0.396 |
|  | Both | 18 (11.7%) | 4(13.8%) | 14(11/2%) | 0.696 |
| Comorbidity | |  |  |  |  |
|  | Pulmonary TB | 89 (57.8%) | 13(44.8%) | 76(60.8%) | 0.120 |
|  | Bronchial tuberculosis | 3 ( %) | 0(0.0%) | 3(2.4%) | 0.999 |
|  | Tuberculous lymphadenitis | 13 ( %) | 2(6.9%) | 11(8.8%) | 0.740 |
|  | Tuberculous meningitis | 4 ( %) | 0(0.0%) | 4(3.2%) | 0.999 |
|  | Milliary TB | 7 ( %) | 1(3.4%) | 6(4.8%) | 0.754 |
| Clinical Chemistry (pleural effusion) | |  |  |  |  |
|  | Total Protein (g/L) | 48.5±7.2 | 49.8±8.4 | 48.3±7.0 | 0.517 |
|  | Total Bilirubin (mmol/L) | 8.6±5.6 | 10.7±9.7 | 8.3±4.7 | 0.208 |
|  | Adenosine deaminase (U/L) | 60.3±28.8 | 60.6±33.8 | 60.3±28.2 | 0.969 |
|  | Glucose (mmol/L) | 3.3±1.5 | 2.6±1.5 | 3.4±1.5 | 0.094 |
|  | Lactate dehydrogenase (U/L) | 876.7±642.8 | 1062.1±1047.2 | 848.8±563.8 | 0.317 |
|  | Amylase (U/L) | 29.6±10.4 | 26.8±11.1 | 30.0±10.3 | 0.345 |
| Blood analysis | |  |  |  |  |
|  | White blood cell (10^9^/L) | 7.3±2.6 | 6.9±2.2 | 7.4±2.7 | 0.374 |
|  | Red blood cell (10^12^/L) | 4.4±0.5 | 4.5±0.5 | 4.4±0.5 | 0.382 |
|  | Hemoglobin (g/L) | 121.2±14.2 | 124.7±16.5 | 120.4±13.5 | 0.157 |
|  | Hematocrit | 36.6±3.9 | 37.4±4.2 | 36.4±3.9 | 0.234 |
|  | Mean corpuscular volume (fL) | 82.4±5.1 | 82.9±5.7 | 82.3±4.9 | 0.580 |
|  | Mean corpuscular haemoglobin (pg) | 27.3±2.1 | 27.7±2.4 | 27.3±2.1 | 0.368 |
|  | Mean corpuscular haemoglobin concentration (g/L) | 331.3±12.7 | 333.1±11.4 | 330.9±13.0 | 0.417 |
|  | Platelet (10^9^/L) | 352.8±129.0 | 322.8±101.1 | 359.7±134.1 | 0.173 |
|  | Neutrophil (10^9^/L) | 5.1±6.2 | 4.3±1.8 | 5.3±6.8 | 0.345 |
|  | Lymphocyte (10^9^/L) | 1.7±0.9 | 1.7±0.6 | 1.7±1.0 | 0.851 |
|  | Monocyte (10^9^/L) | 0.8±0.4 | 0.6±0.3 | 0.8±0.4 | 0.082 |
|  | Coefficient of variation of red cell distribution width (%) | 13.9±1.8 | 13.8±1.6 | 13.9±1.9 | 0.803 |
| Flow cytometry | |  |  |  |  |
|  | CD19+ (%) | 24.8±20.1 | 21.8±19.4 | 25.3±20.4 | 0.612 |
|  | CD3+ (%) | 62.4±13.7 | 62.2±12.2 | 62.4±14.0 | 0.962 |
|  | CD3+CD4+ (%) | 33.1±9.1 | 36.2±4.6 | 32.6±9.6 | 0.259 |
|  | CD3+CD8+ (%) | 24.0±12.0 | 20.7±10.4 | 24.6±12.2 | 0.344 |
|  | CD3-CD16+CD56+ (%) | 11.8±6.2 | 12.5±5.0 | 11.7±6.4 | 0.686 |
|  | CD4+/CD8+ | 2.8±3.9 | 2.2±1.3 | 2.9±4.2 | 0.623 |
| TB, tuberculosis; OR, odds ratio; CI, confidence interval. | | | | | |
